# The prevalence of selected risk factors for non-communicable diseases in Hargeisa, Somaliland: a cross-sectional study

**DOI:** 10.1186/s12889-019-7101-x

**Published:** 2019-07-04

**Authors:** Soheir H. Ahmed, Haakon E. Meyer, Marte K. Kjøllesdal, Niki Marjerrison, Ibrahimu Mdala, Aung Soe Htet, Espen Bjertness, Ahmed A. Madar

**Affiliations:** 1 College of Medicine & Health Science, University of Hargeisa, Hargeisa, Somaliland; 2 Department of Community Medicine and Global Health, Institute of Health and Society, University of Oslo, Oslo, Norway; 3 Division of Mental and Physical Health, Norwegian Institute of Public Health, Oslo, Norway; 4 International Relations Division, Ministry of Health and Sports, Nay Pyi, Taw, 15011 Myanmar

**Keywords:** STEPs survey, Prevalence, Non-communicable disease risk factors, Somaliland

## Abstract

**Background:**

Non-communicable diseases (NCDs), particularly cardiovascular diseases, diabetes, respiratory conditions and cancers, are the most common causes of morbidity and mortality globally. Information on the prevalence estimates of NCD risk factors such as smoking, low fruit & vegetable intake, physical inactivity, raised blood pressure, overweight, obesity and abnormal blood lipid are scarce in Somaliland. The aim of this study was to determine the prevalence of these selected risk factors for NCDs among 20–69 year old women and men in Hargeisa, Somaliland.

**Methods:**

A cross-sectional study was conducted in five districts of Hargeisa (Somaliland), using the STEPwise approach to noncommunicable disease risk factor surveillance (STEPS) to collect data on demographic and behavioral characteristics and physical measurements (*n* = 1100). The STEPS approach is a standardized method for collecting, analysing and disseminating data on NCD risk factor burden. Fasting blood sugar, serum lipids (total cholesterol, low-density lipoprotein (LDL), high-density lipoprotein (HDL), and triglycerides) were collected in half of the participants.

**Results:**

The vast majority of participants had ≤1 serving of fruits daily (97.7%) and ≤ 1 serving of vegetables daily (98.2%). The proportion of participants with low physical activity levels was 78.4%. The overall prevalence of high salt intake was 18.5%. The prevalence of smoking and khat chewing among men was 27 and 37% respectively, and negligible among women. In women, the prevalence of hypertension increased from 15% in the age group 20–34 years to 67% in the age group 50–69 years, the prevalence of overweight and obesity (BMI ≥ 25 kg/m^2^) from 51 to 73%, and the prevalence of diabetes from 3 to 22%. Similar age-trends were seen in men.

**Conclusion:**

Most of the selected risk factors for noncommunicable diseases were high and increased by age in both women and men. Overweight and obesity and low physical activity needs intervention in women, while hypertension and low fruit and vegetable consumption needs intervention in both men and women. Somaliland health authorities should develop and/or strengthen health services that can help in treating persons with hypertension and hyperlipidaemia, and prevent a future burden of NCDs resulting from a high prevalence of NCD risk factors.

## Background

Epidemiological and demographic transitions are in progress in low- and middle-income countries [1], leading to an increasing burden of non-communicable diseases (NCDs). According to the World Health Organization (WHO), the main types of NCDs are cardiovascular diseases (CVDs), cancer, chronic pulmonary obstructive disease (COPD) and diabetes [2]. These NCDs caused 39.8 million global deaths in 2015 [3]. Nearly three-quarters of all NCDs deaths and the majority of premature deaths occur in low- and middle-income countries and before the age of 70 years [4]. NCDs such as CVDs are a primary health concern and a major cause of morbidity and mortality worldwide [5].

Behavioural and metabolic risk factors both contribute significantly to NCDs. They are often interrelated and include unhealthy diet, insufficient physical activity, smoking, excessive use of alcohol, raised blood pressure, overweight and obesity, and abnormal blood lipid levels [6, 7]. Raised blood pressure, dyslipidaemia and smoking account for the majority cause of heart attack and strokes [8, 9]. Further, sociodemographic factors such as age, gender and education have been associated with increased NCDs risk [10].

Somaliland (formerly North Somalia) has a population estimated at 4.5 million people, of which approximately 53% resides in urban areas [11]. Health indicators for Somaliland are among the worst in Sub-Saharan Africa (SSA), with key issues being related to poor governance and inadequate financial resources [12]. Data about NCDs and their risk factors in this region are scarce. We have previously reported that the prevalence of overweight and obesity was significantly higher among Somalis in Oslo, Norway compared to Hargeisa, Somaliland [13], and according to the Ministry of Health of Somaliland, unhealthy lifestyle, including physical inactivity, smoking and chewing khat, has led to an increase in NCDs [14]. Urbanization has been identified as a root cause of the increasing prevalence of NCDs and their risk factors in sub-Saharan Africa [15]. In Hargeisa, the urban society might have adopted a new sedentary lifestyle and the return of the diaspora might bring negative aspects of Western lifestyles, increasing the population’s risk of NCDs [14]. Insight into the prevalence of selected risk factors for NCDs in Somaliland is important, as the country is currently undergoing rapid urbanization and epidemiological transitions [16, 17]. A rise in NCDs and their risk factors will add a high burden to an already pressed health system due to infectious diseases. The aim of the present study is to determine the prevalence of selected risk factors for NCDs among 20–69 year old women and men in Hargeisa, Somaliland.

## Methods

### Study design and sampling

A cross-sectional study was carried out in Hargeisa (capital of Somaliland) according to the WHO STEPwise approach to chronic disease risk factor surveillance [18], between March and September 2016. The STEPS approach is a standardized method for collecting, analysing and disseminating data on NCDs and their risk factors’ burden [18]. We included all three STEPS: STEP (1) Questionnaire survey, including the socio-demographic characteristics, behaviours and dietary habits, and history of hypertension and diabetes; STEP (2) Physical measurements, including anthropometric measurements and blood pressure, and STEP (3) A laboratory investigation of fasting blood glucose and fasting lipid profiles. The STEPS survey has not been validated among Somalis, but it has been pilot tested in the study setting.

Before the inception of the study, two study teams were selected and underwent three days of training before the study implementation. A pilot study was conducted in 20 households and it did not lead to any modification of the questionnaire or measurement procedures (weight, height, waist and hip circumference and blood pressure). The selection criteria were women and men aged 20–69 years. Exclusion criteria were confirmed pregnancy or those diagnosed with terminal or incapacitating illnesses.

Due to the lack of data on the prevalence of the selected risk factors on the population under study, the sample size was calculated using the diabetes prevalence of 4% [19]. There is no population registry in Somaliland, and the only available registry is the number of households. Each household has a unique number. Hargeisa city composes of five major districts, of which each district is further subdivided into four main subdistricts. The sample design for the survey was two-stage cluster sampling and the 20 subdistricts were the primary sampling unit (PSU) (first stage). Out of the twenty PSUs, ten were randomly selected and at the second stage, 1100 households were randomly selected from the ten subdistricts based on the probability proportionate to size (PPS) in each subdistrict.

In each selected household, all eligible individuals aged 20–69 years living in the house were listed in a Kish grid [20]. The Kish method addresses the selection of gender and different age groups in the sample. Men and women were listed in order of decreasing age and given a rank number. If the selected person rejected participation, the next person on the Kish grid was selected until there was one person from each household participating in the study. If there was nobody at home on the day of the study, a notification letter was left at the door, and we returned the next day until we had a participant from each house. None of the randomly selected households were empty or refused to participate in the study and we included one person from each of the randomly selected 1100 households. A total of 195 eligible men were selected. However, 50 men either refused or could not meet after several contacts, resulting in 74.3% (145/195) of the invited men to be included in the study. Among women only three refused to participate, which means 99.7% (955/958) of those invited then participated.

### Data collection and measurements

Participants were involved in the study for two days: day one for STEP 1 and 2 (questionnaire, anthropometric and blood pressure measurements), and day two for STEP 3 (laboratory tests).

Step 1 involved the survey questionnaire consisting of core information (age, sex, marital status and education) and behavioural variables (fruit and vegetable intake, physical activity, smoking and khat). Medical history included questions on medication for raised blood pressure, diabetes and raised cholesterol.

Step 2 involved physical measurements including weight, height, waist circumference and blood pressure. Weight and height were measured with participants standing without shoes and wearing light clothing. Body weight (kilograms) was recorded to the nearest 0.1 kg and measured with an Omron medical scale that was checked every day with a known weight. Height (centimetres) was recorded to the nearest 0.5 cm and measured with a manual height-measuring instrument (SECA stadiometer) with participants standing upright with the head in Frankfort plane. Body mass index (BMI) was calculated as weight in kilograms divided by the square of the height in meters (kg/m^2^). Waist circumference (WC) was measured at the midpoint between the lower margin of the last palpable rib and the top of the iliac crest, using a measuring tape to the nearest 0.1 cm with the subject standing and breathing normally. Blood pressure was measured 3 times at one minute intervals using an automatic, validated device (Omron HBP 1300) [21], with participants seated and after resting for approximately 5 min.

Step 3 involved a laboratory test. Fasting venous blood samples were collected from participants to determine the concentration of serum glucose and lipids (fasting serum glucose (FSG), total cholesterol (TC), high-density lipoprotein (HDL) cholesterol, low-density lipoprotein (LDL) cholesterol, and triglycerides (TG)). In Hargeisa Group Hospital, blood samples were collected in serum-separator gel tubes and centrifuged after 30 min. Serum and plasma were then separated and frozen in aliquots at − 30 °C the same day until being shipped to Oslo, Norway where they were analysed as one batch at the Fürst Medical Laboratory (http://www.furst.no/), which has been accredited by the Norwegian Accreditation according to the standard NS-EN ISO 15189 TEST 209. The inter-assay coefficients of variation were 1.3% (TC), 1.6% (LDL-cholesterol), 1.8% (HDL-cholesterol), and 3.8% (TG). Total cholesterol, LDL-cholesterol, HDL-cholesterol, and TG were measured using an enzymatic method (ADVIA 2400 Siemens).

### Variables

We classified age (years) into three groups for women (20–34, 35–49 and 50–69) and for men into two groups due to a smaller number of participants (20–34 and 35–69). Overweight and obesity was defined as BMI ≥25 kg/m^2^ [22]. High waist circumference was classified according to the European cut-off (> 94 cm for men, > 80 cm for women) because cut-offs for SSA were not available [23]. Hypertension was defined as systolic blood pressure (SBP) ≥140 mmHg, diastolic blood pressure (DBP) ≥90 mmHg, and/or being on blood pressure-lowering medication [4].

Diabetes was defined as FSG ≥ 7.0 mmol/l and/or being on medication [23], and high cholesterol level as TC/HDL ratio ≥ 5.0 and/or being on medication, and high triglycerides as TG ≥1.7 mmol/l [18, 24].

High salt intake was defined as answering often or always to at least one of the questions regarding salt intake (adding salt to the plate after food has been served, adding salty seasoning or a salty sauce, and eating processed food high in salt). Respondents were asked for the number of days they ate fruit and vegetables in a typical week. Servings were measured by showing pictorial show-cards. A low fruit and vegetables consumption was defined as < 3 portions of fruit and vegetables per day, due to a low number of participants consuming > 5 servings.

Physical activity was measured by asking the respondents about their weekly and daily vigorous and moderate activities during work, leisure time and during transport, and the time spent in these activities. Low physical activity was defined as achieving less than 600 MET-minutes per week in accordance with WHO guidelines, determined by the intensity of exercise undertaken (MET level) multiplied by the minutes participated per week (minutes) [25].

### Statistical methods

Descriptive statistics in the form of frequencies (proportions) and means with standard deviations (SD) were used to summarize the data. Associations between categorical variables were established from the Chi-square test and/ or the Fishers’ exact test. The Chi-square test was used to assess the association between each categorical variable with gender. In particular, we used a Chi-square test to investigate whether there was a significant increase (trend) of each risk factor across the different age groups (a variable with a natural ordering). Age-specific means stratified by gender and age-groups were estimated by using one-way analysis of variance (ANOVA) to test for the differences between the means. All statistical analyses were performed using SPSS version 25.0 (Chicago, Illinois: SPSS Inc.), and differences with a *p*-value < 0.05 were considered statistically significant.

## Results

### Demographic and lifestyle characteristics

A total of 1100 participants were included in the analysis, with a higher proportion of women (*n* = 995) than men (*n* = 145). All participants underwent anthropometric measurements, while blood samples were drawn from 597 participants (56.2% of women and 41.3% of men). The majority of participants were in the age group 20–34 years (43.3%) (Table 1). Among women, 66.4% had no formal education; the corresponding proportion among men was 31.7%. More than two-thirds of the participants were married. Almost all participants had a low fruit and vegetables consumption, physical activity (overall MET < 600 min/week) was reported among 81.5% of women and 58.0% of men. The overall proportion with a high salt intake was 18.5%. Khat chewing (36.6%) and smoking (26.9%) was common among men, whereas only 0.3% of women chewed khat and none smoked (Table 1).

**Table 1:** Characteristics of study participants by gender

	Women(*n* = 955) N (%)	Men (*n* = 145) N (%)	Total (*n* = 1100) N (%)	*P*-value*
Age (years)				
20–34	402 (42.5)	76 (52.4)	478 (43.5)	0.015
35–49	290 (30.4)	28 (19.3)	318 (28.9)	
50–69	263 (27.5)	41 (28.3)	304 (27.6)	
Marital status				
Never married	165 (17.2)	60 (41.4)	224 (20.4)	< 0.001
Currently married	749 (78.4)	82 (56.6)	831 (75.5)	
Divorced	359 (3.7)	2 (1.4)	37 (3.4)	
Widowed	6 (0.6)	1 (0.7)	0.6 (7)	
Education				
No education	634 (66.4)	46 (31.7)	680 (61.8)	< 0.001
Primary education	185 (19.4)	23 (15.9)	208 (18.9)	
Secondary education	74 (7.7)	31 (21.4)	105 (9.5)	
University education	62 (6.5)	45 (31.0)	107 (9.7)	
Fruit consumption				
No servings/day	240 (25.1)	24 (16.6)	264 (24.0)	0.083
1 servings/day	695 (72.8)	116 (80.0)	811 (73.7)	
2 servings/day	8 (0.8)	3 (2.1)	11 (1.0)	
3 servings/day	12 (1.3)	2 (1.4)	14 (1.3)	
Vegetable consumption				
No servings/day	270 (28.3)	36 (24.8)	306 (27.8)	0.095
1 servings/day	669 (70.1)	105 (72.4)	774 (70.4)	
2 servings/day	4 (0.4)	3 (2.1)	7 (0.6)	
3 servings/day	12 (1.3)	1 (0.7)	13 (1.2)	
Fruit and vegetables consumption				
< 3 servings/day	930 (97.4)	138 (95.2)	1068 (97.1)	0.140
> 3 servings/day	25 (2.6)	7 (4.8)	32 (2.9)	
Physical activity, MET-min				
> 600	176 (18.5)	60 (42.0)	236 (21.6)	< 0.001
< 600	775 (81.5)	83 (58.0)	858 (78.4)	
Salt^a^				
Often/always	171 (17.9)	32 (22.1)	203 (18.5)	0.233
Others (never & rarely)	782 (82.1)	113 (77.9)	895 (81.5)	
Current smoker				
Yes	–	39 (26.9)	39 (3.5)	< 0.001
No	955 (100)	93 (73.1)	1045 (96.5)	
Current khat chewers				
Yes	3 (0.3)	53 (36.6)	56 (5.1)	< 0.001
No	948 (99.7)	92 (63.4)	1040 (94.9)	

* *P*-value of the *X*^*2*^ test for differences between women and men. ^a^Combined all types of salt intake (added salt, salt sauces and processed food high in salt)

### Risk factor profile across age groups

**Fig. 1:**
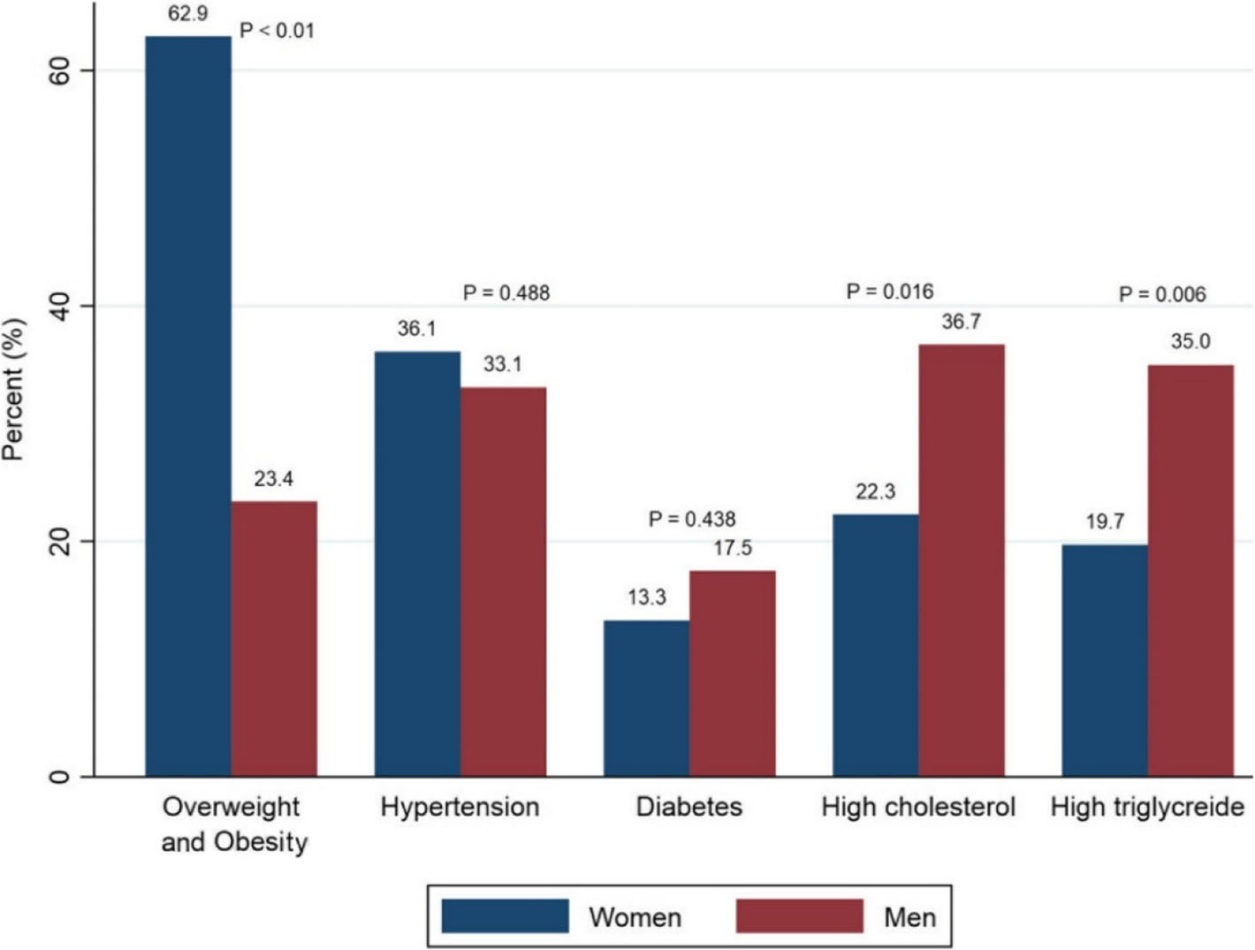
Proportion of NCDs risk factors by gender (*P* value for the gender differences)

We observed differences in risk factors levels across age groups. Among women, mean levels of BMI, WC, SBP, DBP, FSG, TG, LDL and TC to HDL ratio increased with age (Table 2). Over all, women had a high proportion of overweight and obesity (62.9%) compared to men (23.4%), while women (36.1%) and men (33.1%) and had a similar proportion of hypertension. Both high cholesterol and high triglycerides were higher in men (36.7% and 35.0) than in women (22.3 and 19.7%, respectively) (Fig. 1).

**Table 2:** Age- and gender-specific distribution of mean (SD) anthropometric and biochemical risk factors

	Women (*n* = 955)^a^				Men (*n* = 145)^a^		
Age (years)	20–34 years, Mean ± SD	35–49 years, Mean ± SD	50–69 years, Mean ± SD	*p*-value	20–34 years, Mean ± SD	35–69 years, Mean ± SD	*p*-value
BMI(kg/m^2^)	25.5 ± 5.6	28.6 ± 5.9	28.3 ± 5.6	< 0.001	20.8 ± 4.5	23.5 ± 4.2	< 0.001
WC (cm)	81.4 ± 13.6	91.2 ± 13.4	91.2 ± 13.4	< 0.001	75.0 ± 11.4	89.5 ± 11.5	< 0.001
SPB (mmHg)	115.8 ± 13.5	128.7 ± 21.4	146.5 ± 29.1	< 0.001	123.4 ± 22.0	136.7 ± 31.8	0.004
DBP (mmHg)	78.9 ± 9.1	85.8 ± 12.4	89.2 ± 14.2	< 0.001	79.0 ± 10.1	86.8 ± 14.0	< 0.001
FSG (mmol/L)	4.8 ± 1.4	5.6 ± 2.6	5.9 ± 2.9	< 0.001	4.9 ± 1.7	5.8 ± 2.1	0.073
TC (mmol/L)	4.4 ± 1.0	4.6 ± 0.9	5.1 ± 0.8	< 0.001	4.2 ± 1.3	4.8 ± 0.9	0.053
HDL (mmol/L)	1.1 ± 0.3	1.1 ± 0.3	1.1 ± 0.3	0.457	0.9 ± 0.3	1.0 ± 0.2	0.127
TC to HDL ratio	3.9 ± 1.0	4.2 ± 1.1	4.5 ± 0.9	< 0.001	4.3 ± 0.9	4.7 ± 0.9	0.118
Triglyceride (mmol/L)	1.0 ± 0.5	1.4 ± 0.6	1.6 ± 0.9	< 0.001	1.5 ± 1.9	1.6 ± 0.7	0.739
LDL (mmol/L)	2.5 ± 0.9	2.8 ± 0.9	3.1 ± 1.0	< 0.001	2.3 ± 1.1	3.0 ± 0.8	0.004

^a^n is lower among those who did blood sample (women *n* = 537, men = 60)

Abbreviations: *BMI* Body mass index, *WC* Waist circumference, *SBP* Systolic blood pressure, *DBP* Diastolic blood pressure, *FSG* Fasting serum glucose, *TC* Total cholesterol, *HDL* High-density lipoprotein, *LDL* Low density lipoprotein. TC to HDL ratio: total cholesterol to High-density lipoprotein

Further, the prevalence of hypertension, overweight and obesity, high WC, diabetes, high TG and high cholesterol increased with age (Table 3). Only a high salt intake was higher in younger compared to older women (26.9% vs 13.3%).

**Table 3:** Age- and gender-specific prevalence of behavioural, physical and biochemical risk factors

Women (*n* = 955)^**£**^				
Risk factor	20–34 years, % (95%CI)	35–49 years, % (95%CI)	50–69 years, % (95%CI)	Pvalue^*^
Low fruit and vegetables consumption	97.5 (95.9,99.0)	96.2 (94.0,98.4)	98.5 (98.5,96.9)	0.242
High salt intake	26.9 (23.0,31.0)	9.7 (6.0,13.0)	13.3 (9.0,17.0)	< 0.001
Low physical activity	81.0 (77.1,84.8)	78.5 (73.8,83.8)	85.6 (81.3,89.8)	0.103
Hypertension	14.8 (11.0,17.9)	37.8 (32.2,43.4)	67.2 (61.4,72.9)	< 0.001
Overweight and obesity	50.7 (46.0,56.0)	71.0 (66.0,76.0)	72.6 (67.0,78.0)	< 0.001
High waist circumference	51.6 (47.0,57.0)	81.4 (77.0,86.0)	82.1 (77.0,87.0)	< 0.001
Diabetes	3.4 (0.9,5.8)	16.3 (10.8,21.7)	22.2 (15.9,28.5)	< 0.001
High triglycerides	10.7 (6.4,15.0)	21.8 (15.5,28.0)	29.0 (21.9,36.0)	< 0.001
High cholesterol	13.6 (8.8,18.3)	24.1 (17.6,30.6)	31.6 (24.3,38.9)	< 0.001
Men (*n* = 145) ^**£**^				
Risk factor	20–34 years,% (95%CI)	35–69 years,% (95%CI)	NA	^*^Pvalue
Low fruit and vegetables consumption	98.7 (98.7,99.1)	91.3 (84.4,98.1)		0.038
High salt intake	23.7 (14.0,33.0)	20.0 (11.0,30.0)		0.623
Low physical activity	56.6 (45.2,67.9)	59.7 (47.6,71.8)		0.708
Current smoking	18.4 (10.0,27.0)	36.2 (25.0,48.0)		0.016
Current khat chewers	28.9 (19.0,39.0)	44.9 (33.0,57.0)		0.046
Hypertension	17.1 (8.4,25.8)	50.7 (38.6,62.8)		< 0.001
Overweight and obesity	14.5 (16.0,23.0)	33.3 (22.0,45.0)		0.007
High waist circumference	6.6 (1.0,12.0)	40.6 (29.0,52.0)		< 0.001^**^
Diabetes	7.4 (−3.1,17.9)	25.0 (10.1,39.8)		0.097^**^
High triglycerides	25.9 (8.2,43.6)	42.4 (24.6,60.2)		0.189
High cholesterol	20.0 (3.1,36.8)	48.6 (21.8,56.9)		0.031^**^

*chi-square test for trend in proportions, **Fisher’s exact test

^£^ N is lower among those who did blood sample (women *n* = 537, men = 60)

Low fruit and vegetables consumption (< 3 servings of fruits and/or vegetables on average per day), high salt intake (answering often and/or always to at least one of the questions regarding salt intake (adding salt to the plate after food has been served, adding salty seasoning or a salty sauce, and eating processed food high in salt)). Low physical activity (< 600 MET-min), current smoking (Yes/No), current khat chewers (Yes/No). Hypertension (SBP ≥140 mmHg, DBP ≥90 mmHg and/or being on blood pressure lowering medication), overweight and obesity (BMI ≥ 25 kg/m^2^). High waist circumference (≥80 cm for women & ≥ 94 cm for men), Diabetes (≥7.0 mmol/l and/or being on medication). High triglycerides level (≥ 1.7 mmol/l). High cholesterol (TC: HDL ratio ≥ 5 and/or being on medication)

Among men, the mean levels of BMI, WC, SBP, DBP, and LDL was higher in the oldest age group than among the younger (Table 2), and the prevalence of smoking, khat chewing, hypertension, overweight/obesity and high WC increased with age (Table 3). A low fruit and vegetables consumption was found in both age groups, but was most pronounced among the youngest men. The prevalence of diabetes ranged from 3.4% in the youngest age group to 22.2% in the oldest among women, and from 7.4 to 25.0% among men. Corresponding proportions for hypertension were 14.8 and 67.2% among women and 17.1 to 50.7% among men. Around two-thirds of women, but less than one-third of men were overweight/obesity and/or had high WC, with the highest proportions for each measure found in the oldest age group.

### The proportion of behavioural risk factors by level of education

We examined educational differences in behaviour risk factors but we found no differences, only that khat chewing was higher among the less educated men compared to those had university education (Table 4).

**Table 4:** Gender distribution of behavioural risk factors by level of education

Education				
	Preschool/Primary	Secondary	University	
Women	(*n* = 819), N (%)	(*n* = 74), N (%)	(*n* = 62), N (%)	Pvalue*
Low fruit and vegetables consumption	795 (97.1)	74 (100)	61 (98.4)	0.280
High salt intake	143 (17.5)	15 (20.3)	13 (21.0)	0.682
Low physical activity	665 (81.4)	61 (83.6)	49 (79.0)	0.796
Men	(*n* = 69), N (%)	(*n* = 31), N (%)	(*n* = 45), N (%)	
low fruit and vegetables consumption	65 (94.2)	30 (96.8)	43 (95.6)	0.188
High salt intake	14 (20.3)	8 (25.8)	10 (22.2)	0.827
Low physical activity	42 (62.7)	19 (61.3)	22 (48.9)	0.320
Current smoking	22 (31.9)	8 (25.8)	9 (20.0)	0.372
Current khat chewers	33 (47.8)	11 (35.5)	9 (20.0)	0.010

*Pvalue for the chi-square test

Low fruit and vegetables consumption (< 3 servings of fruits and/or vegetables on average per day), High salt intake (answering often and/or always to at least one of the questions regarding salt intake (adding salt to the plate after food has been served, adding salty seasoning or a salty sauce, and eating processed food high in salt)). Low physical activity (< 600 MET-min), current smoking (Yes/No), current khat chewers (Yes/No)

## Discussion

This study reported that selected risk factors of non-communicable diseases were prevalent among both women and men in Hargeisa, suggesting a significant threat to the Somali population related to an upcoming NCDs epidemic. For most risk factors, the prevalence increased with age. Smoking and khat chewing were a problem among men only, whereas a higher proportion of women than men reported low levels of physical activity. Overweight and obesity was higher in women and high blood pressure was high in both women and men. Age is an unmodifiable risk factor for chronic diseases across all populations. In our study, age was associated with the increasing prevalence of many of the investigated selected NCD risk factors in both women and men, and our findings are consistent with studies that were conducted among other Sub-Saharan African countries [26, 27].

In general, low fruit and vegetable consumption was highly prevalent among women and men, and it was higher than what has been previously reported in South Africa [28]. High consumption of fruit and vegetables is documented to reduce the risk of coronary heart disease, stroke, obesity and possibly some types of cancer [29]. Accessibility and affordability play important roles in the consumption of fruit and vegetables in some low- and middle-income countries [30]. In Somaliland, a big portion of fruits and vegetables are imported from abroad, and thus their consumption depends on several factors including price and seasonal availability. However, in the Somali methods of food preparation, most vegetables are cooked with sauces and stewing, and thus those cooked vegetables are not considered a vegetable consumption nor are fruits consumed as drinks. Thus, vegetable and fruit intake levels in this study may be underreported.

The high proportion of overweight and obesity among women compared to men has been reported previously [13], and is consistent with other findings [31]. The high prevalence of overweight and physical inactivity in women are likely interrelated [32, 33], as physical inactivity is a risk factor for obesity and other chronic diseases [34]. Cultural norms such as a desire to be overweight to demonstrate wealth, and a lack of social and environmental support, may be leading to reduced participation in physical activity [35]. In some populations, lower physical activity among women has been attributed to gender norms, including gender constraints and a lack of suitable dresses for exercise [36]. Undertaking physical activity in a fitness centre in Hargeisa for women requires gender-separated facilities, which are limited.

Additionally, nearly no women smoked or chewed khat as previously reported [37]. There may be sociocultural explanations for this, as smoking could be seen as a normal part of being a man [38]. Additionally, chewing khat among women in some societies is considered socially unacceptable and thus is rarely used [39]. Meanwhile, one in three men chewed khat and one in four smoked. The crude prevalence of current smoking among men in Hargeisa, at 26.9%, was higher than in Ethiopia, Benin and Ghana, but lower than in Sierra Leone, Lesotho and Madagascar [40]. Such a prevalence is concerning, as smoking alone is a well-established and major risk factor for CVD, cancer and COPD, and is the second leading cause for CVD mortality after high blood pressure [41].

Khat are the fresh leaves that are widely chewed in Yemen and other East African countries, which are chewed for leisure and social purposes [42]. Khat chewing is correlated with various health consequences associated with chronic diseases, such as raised blood pressure and coronary heart disease [43–45]. The high prevalence of khat chewing among men in this study is similar to findings from Ethiopia [46].

Another finding in our study was the higher prevalence of behaviour risk factors like khat and smoking among the older age group of men (35 to 69 years) than the younger (20 to 34 years). Our finding of smoking being higher among the oldest age group is contrary to a previous study conducted among Nigerians [47]. As it is known that smoking and khat chewing are both social habits among men in East African countries, we are unsure if the lower prevalence seen among the younger age group will increase, as they grow older. Little is known about khat chewing in East African countries, and changing trends with the ageing population and rising economy should be further investigated.

We have examined the differences between women and men in overweight and obesity, hypertension and high cholesterol. We found that women had higher overweight and obesity, whereas men had higher high cholesterol than women. However, hypertension was high among both women and men and these metabolic risk factors tend to increase with age. Concerningly, high blood pressure combined with high cholesterol substantially increases CVD risk [48]. The current population can be expected to live longer than previous generations, which will give rise to greater NCD risks among the population and put a greater demand on the health resources of the nation. Coupled with scarce health-care facilities and financial resources for healthcare, a lack of awareness, prevention and treatment of chronic disease risk factors may further increase CVD morbidity and mortality in Somaliland [49].

### Strengths and limitations

In general, data on the prevalence of risk factors for NCDs among Somalis in the Horn of Africa is very limited, and to our knowledge, this is the first study that has been carried out in Somaliland using the WHO STEPwise approach to noncommunicable disease risk factor surveillance (STEPS). This provides important baseline data on the prevalence of risk factors for CVDs for comparison to other Somali regions and for inclusion in follow-up studies of longitudinal design. The tools used in the study were standardized and checked every morning.

This study was carried out in an urban setting. The sample was drawn from a big city with Somali inhabitants originating from all regions throughout the Horn of Africa. Thus, our results could possibly be representative of other cities in Somaliland and Somalia.

This study has some limitations. The low participation of men in this study limits the interpretation of data pertaining to them. In our survey we used the Kish grid, as the Kish grid addresses the selection of gender and age in a sample, but there is a discussion of whether the Kish grid can provide a representative sample for gender [50]. During the survey, more women were in the households than men. Moreover, fifty eligible men refused or were not available after several contacts. If the selected person was not at home, we left a note that our team would come back next day, and if they were not present for two attempts, we selected the next eligible person from the Kish grid. According to Somali culture, women are often in the houses during the daytime while men are away working or socializing with other men. Therefore, there also might have been a selection bias among men who were home and included in the study, as they may have been home and willing to participate for special reasons such as poor health or lack of work. Again, results pertaining to the low number of men in this study should be treated cautiously.

The lower number of participants who participated in the blood sample analysis (54.3%) is also a possible limitation of this study. This might have been due to negative perceptions towards blood sampling in Somali culture or the location of the blood collection was far for some of the participants (health centre). It is possible that those who took the blood tests are those who have a more active awareness of their personal health. However, the BMI, SBP and DBP of the participants who did not take blood samples were not significantly different from the participants in the analysis.

Lastly, recall bias may have been introduced from the questions on the self-reported variables such as that pertaining to vegetable and fruit intake, as it possible that participants didn’t report boiled or cooked vegetables (such as cabbage, tomatoes, carrots, and onions) with main dishes such as rice and spaghetti.

## Conclusion

The prevalence of selected NCDs risk factors are high in Somaliland. Overweight and obesity and low physical activity needs intervention in women, while hypertension and low fruit and vegetable consumption needs intervention in both men and women. Policy makers and stakeholders in the health sector need to institute nationwide population-based strategies to create awareness about these selected NCDs risk factors, as well as its consequences. In addition, more research should be undertaken to generate representative data on NCDs risk factors differences among urban and rural areas, genders, and socio-economic conditions.

## Data Availability

The data was stored and/or analysed at the University of Oslo’s platform for the processing of sensitive research data, TSD (https://www.uio.no/english/services/it/research/sensitive-data/). Data is available on reasonable request.

## Abbreviations

ANOVA: Analysis of variance
BMI: Body mass index
CI: Confidence interval
CVDs: Cardiovascular diseases
MET: Metabolic equivalents
NCDs: Noncommunicable diseases
PSUs: Primary sample units
SSA: Sub-Saharan Africa
WC: Waist circumference
WHO: World Health Organization
